# The influence of inflammation on cardiovascular disease in women

**DOI:** 10.3389/fgwh.2022.979708

**Published:** 2022-10-11

**Authors:** Sita Kottilil, Poonam Mathur

**Affiliations:** ^1^ Yale University, New Haven, CT, United States; ^2^ Insitute of Human Virology, University of Maryland School of Medicine, Baltimore, MD, United States

**Keywords:** women, sex, inflammation, biomarkers, cardiovascular disease risk

## Abstract

The onset of cardiovascular disease in women is almost a decade later than men, partly due to the protective effect of estrogen prior to menopause. Recently, it was noted that while there have been advances in improving the morbidity and mortality from CVD in women older than 55 years, the improvement in younger women has been stagnant. The mechanism behind this lag is unclear. This manuscript reviews the literature available on the sex-specific inflammatory response in the context of traditional and non-traditional cardiovascular disease risk factors. Our review suggests that women have a differential inflammatory response to various disease states that increases their risk for CVD and warrants a distinct prioritization from men when calculating cardiovascular disease risk.

## Introduction

Cardiovascular disease (CVD) is the leading cause of mortality globally and among women (1). Though CVD-related mortality for women has decreased over the last four decades, the improvement has been seen mainly in older women (>55 years), with stagnant mortality rates in younger women (2). The reason for this discrepancy is unclear, since the pre-menopausal state of younger women is protective against CVD in women, such that there is an approximate 10-year lag for the first atherosclerotic cardiovascular event in women (3). One theory for this is that monthly iron loss prior to menopause is cardioprotective compared to men (4). However, the more widely supported theory for why CVD- related events increase after menopause is that estrogen has been shown to have an anti-inflammatory effect (5), and the estrogen loss associated with menopause attributes to the delayed onset of CVD in women.

However, since CVD-mortality rates in younger women have remained static, there are likely etiologies not related to estrogen or other hormones that play a role in CVD risk. In addition, since men have not had stagnant rates of CVD-related mortality, it is important to understand if there are sex-specific differences that affect how traditional and non-traditional cardiac risk factors actually affect CVD risk. Studies have shown that women with higher risk for CVD also have higher rates of the traditional cardiac risk factors (3), such as diabetes mellitus, hypertension, and obesity (6). In addition to the aforementioned factors, smoking, physical inactivity, and dyslipidemia are also acknowledged as traditional risk factors that increase CVD risk in women.

Over the last 20 years, non-traditional risk factors, including adverse pregnancy outcomes, autoimmune diseases, breast cancer treatment, and depression have been associated with increased CVD risk (7). Another significant risk factor is systemic inflammation (8). Inflammation's role in the pathogenesis of CVD ranges from the initiation of atherosclerosis to subsequent plaque formation, plaque rupture, and ultimately, precipitation of thrombosis (9). Inflammatory markers, especially C-reactive protein (CRP), are acute-phase reactants that are markers of inflammation in the body and are associated with higher risk of CVD and predict future cardiac events (10–13). In healthy middle-aged and older women, baseline levels of CRP are independent predictors of CVD (14). In addition, CRP levels have been shown to be elevated in women compared to men, whether atherosclerotic risk factors are present or not, even after accounting for hormone replacement therapy (15–17). In a study of 2,219 adults in the US who had acute myocardial infarction, women younger than age 55 had higher levels of circulating CRP compared to men (18). The higher level of CRP was only partially explained by a higher prevalence of smoking, diabetes mellitus, and obesity in that group. In addition, in a sub-analysis of 7,184 patients from the Framingham Heart Study, cardiac biomarkers, including CRP, were significantly higher in pre-menopausal women than men, and this difference was attenuated in post-menopausal women who were not on hormone replacement therapy (19).

It is possible that other inflammatory biomarkers, such as interleukins (ILs) and Tumor Necrosis Factor (TNF) play a role in predisposing to CVD risk, and that sex-specific differences in CVD risk are associated with these biomarkers. Therefore, in this manuscript, we will review the literature that identifies sex-specific differences in the association between inflammatory biomarkers and traditional and non-traditional cardiac risk factors. Due to the lack of improvement seen in CVD-related morbidity and mortality in younger (pre-menopausal) women, we will not be reviewing how estrogens and other hormones play a role in mitigating CVD risk. Rather, we will focus on those human studies that directly link discrepancies between women and men in inflammatory biomarkers with risk factors for CVD, so that there may be some explanation for the CVD risk that has remained in younger women, and how this gap may be explained prior to the significant hormonal changes that occur with the onset of menopause.

**Figure 1:**
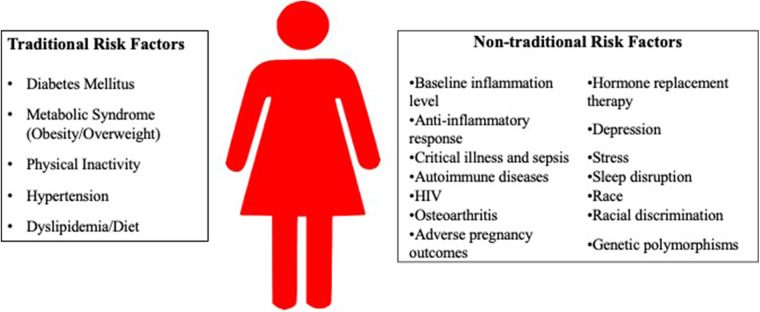
Risk factors for cardiovascular disease in women.

Of note, sex differences are a result of differences in gene expression from sex chromosomes, and gender differences are a result of individual experience and sociocultural practices. However, to facilitate discussion of sex-specific differences in inflammation that contribute to traditional and non-traditional CVD risk factors, “woman” or “women” will be used in this manuscript, since many studies examining sex differences use this terminology. A summary of the risk factors is found in Figure 1, and Table 1 summarize the inflammatory markers associated with each risk factor.

**Table 1:** Female-specific inflammatory conditions that predispose to CVD.

**Traditional Risk Factors**	**Levels of inflammatory markers in women compared to men**
Diabetes Mellitus	↑ CRP
	↑ TNF-α
	↑ IL-6
Smoking	↓ CRP
Metabolic Syndrome/Obesity, Overweight	↑ CRP
	↑ IL-1RA
	↑ IL-8
	↑ IL-6
Physical inactivity	Discordant findings for CRP
Hypertension	↑ CRP
Dyslipidemia/Diet	↑ CRP
	↑ adiponectin
**Non-traditional Risk Factors**	
Baseline inflammation level	↑ MCP
	↑ IL-12
	↑ CRP
Anti-inflammatory response	↓ IL-10
Critical illness and sepsis	↑ CRP
Autoimmune diseases	↑ ESR
HIV	↑ CRP
Osteoarthritis	↑ CRP
Adverse pregnancy outcomes	More data needed
Hormone replacement therapy	↑ CRP
Depression	↑ CRP
	↑ IL-6
Stress	↑ IL-6
	↑ MCP-1
	↓ CRP
Sleep disruption	↑ CRP
	↑ IL-6
	↑ TNF-α
Race	↑ CRP
Racial discrimination	↑ CRP
Genetic polymorphisms	↑ CRP

CRP, C-reactive protein; TNF-a, Tumor Necrosis Factor alpha; IL, Interleukin; IL-1RA, IL-1 receptor antagonist; MCP, monocyte chemoattractant protein; ESR, Erythrocyte sedimentation rate.

### Traditional risk factors

#### Diabetes

Women with diabetes have a higher relative risk of incident CVD than their male counterparts. This is in part related to sex differences in inflammation that predispose to diabetes, depicted by three studies. Development of diabetes and insulin resistance have been associated with higher expression of inflammatory cytokines by adipose tissue, including CRP (20, 21), TNF-*α*, IL-1, and IL-6 (22). CRP as a risk factor for the 6-year development of diabetes mellitus and the metabolic syndrome were evaluated in the Mexico City Diabetes Study (23). At baseline, CRP correlated significantly (*p* < 0.001) with all metabolic indexes in women (dyslipidemia, hypertension, and diabetes), but less so than in men. After a 6-year follow-up, women with the highest CRP values (stratified into tertiles), had an increased relative risk of developing metabolic syndrome and diabetes, and the risk changed minimally after adjusting for Body Mass Index (BMI) and insulin resistance. Second, a study by Macarenhas-Melo et al., who investigated the risk for CVD after menopause found that in women, diabetes compounded the CVD risk of postmenopausal women compared to men who are diabetic and matched for age (24). The worsening CVD risk was associated with higher levels of CRP and TNF-*α* compared to men. Last, in a large, prospective cohort study in the United States, more than 27,000 middle-aged women without diabetes or CVD were followed for 4 years and compared to disease-free controls (25). Baseline levels of IL-6 and CRP were significantly (*p* < 0.001) higher among cases than controls. The relative risks for future DM for women were 7.5 for IL-6 and 15.7 for CRP. These associations persisted after controlling for BMI, family history of diabetes, smoking, exercise, alcohol use, and hormone replacement therapy. These studies, though few, suggest that CRP and IL-6 contribute to increased CVD risk in women with diabetes.

#### Smoking

Smoking increases levels of circulating inflammatory cytokines including CRP, IL-6, and TNF-*α* (26, 27). In a study exclusively of sedentary female smokers ages 18–55, even if smoking cessation was not achieved, a reduction in tobacco exposure and increase in exercise (measured by oxygen consumption) significantly reduced inflammation, as seen by a decrease in circulating white blood cells (28). However, there was no significant reduction in CRP, suggesting that in women, smoking creates long-lasting increases in systemic inflammation that can increase CVD risk. However, a randomized control trial of men and women suggests that smoking does not have as significant of a role on underlying inflammation in women as it does for men (29). However, the study did not evaluate the outcomes associated with second-hand smoke, so it is unclear if sexual dimorphism affects clinical outcomes due to smoking- induced inflammation. There is limited data available on the sex differences of smoking on inflammation, so more studies must be done to determine if this traditional risk factor increases CVD risk in women more than men *via* increased systemic inflammation.

#### Metabolic syndrome / obesity and overweight

One of the most influential pre-existing risk factors for CVD, metabolic syndrome, includes hypertension, elevated fasting glucose, elevated triglycerides, low serum HDL, and abdominal obesity. Metabolic syndrome is a strong predictor of CVD in women, moreso than men (30). This is seen in multiple ethnic groups, including Chinese women (31). In women who meet all criteria for metabolic syndrome, there is an exaggerated inflammatory response compared to men, further increasing the CVD risk, and this response predicts the development of the metabolic syndrome more accurately than for men (32). A study by Ahonen et al. investigated Finnish subjects with metabolic syndrome and found men had lower CRP and IL-1RA levels compared to women (33). In addition, a large cross-sectional study of 3,037 subjects over 7 years showed that CRP levels were elevated in women with metabolic syndrome and were strongly related to each component of metabolic syndrome (34). There are also differences in how the hormones that protect against metabolic syndrome affect inflammation. In healthy women, the hormone adiponectin is associated with favorable lipid profiles. In women with renal transplants, there is a significant negative correlation between adiponectin levels and serum CRP in women, suggesting a reduced risk of CVD (35). In the same study, however, men with CVD had a significant positive correlation between adiponectin levels and baseline CVD. Therefore, even hormones that play a role in the development of metabolic syndrome have varying effects on inflammation between the sexes.

Metabolic syndrome's effect on CVD risk is compounded in patients with peripheral arterial disease, moreso in women than men, who have increased levels of inflammatory biomarkers such as IL-8, leptin, and pigment epithelium-derived factor (36). Also, women with central obesity have elevated oxidative stress markers (CRP) compared to men with central obesity and thus have a higher risk than men for CVD (37).

Obesity is a component of metabolic syndrome. Since there are differences in fat distribution between men and women, the relationship between obesity and CRP also differs. Obesity more greatly impacts women's CVD risk and incidence of CVD compared to men (7). A large, national sample of adults >50 years of age in the United States found that women had a stronger positive association between BMI and higher CRP value (*p* < 0.001) compared to men (38). Unlike other studies, there was no difference between Black women and White women in terms of association of BMI and CRP. Another study of 733 women without preexisting CVD found that both CRP and IL-6 correlated with BMI (39). This association was thought to be secondary to adipose tissue secreting IL-6, which leads to CRP production from the liver (39).

Numerous studies further link obesity and increased BMI to increased CVD risk. The Dallas Heart Study measured CRP in white and black subjects ages 30 to 65 and compared levels of CRP between different race and gender groups (40). After adjustment for traditional cardiovascular risk factors, estrogen and statin use, and body mass index, Black subjects and women had significantly higher levels of CRP than White subjects and men, respectively. In addition, though an increasing BMI was associated with higher CRP levels in each race and gender, the increase in CRP levels with obesity was greater for women than men (*p* < 0.001). In a large Korean cross-sectional study of 18,610 subjects ≥20 years of age, BMI and waist circumference were significantly associated with elevated CRP levels (>3 mg/L) in women but not men (41). Investigators in the CARDIA study found that elevated CRP levels (>10 mg/L) were more likely to be a chronic finding (i.e. more than one measurement of elevated CRP) in women with high BMIs than men with high BMIs (42). In a cross-sectional analysis of over 27,000 apparently healthy US women (mean age 54.7 years), from the Women's Health Study (1992–1995), high BMI was more strongly correlated with adverse cardiovascular biomarker levels (including CRP) than physical inactivity (43). In a study of 795 men and 827 women aged 60 to 64 years from the Medical Research Council National Survey of Health and Development (44), less sedentary time was associated with more favorable levels of CRP and IL-6, and this association was stronger in women than in men. This difference was explained by greater fat mass of women for a given body mass index. Finally, in a cohort of people with HIV(PWH) on antiretroviral therapy, higher BMI and excessive visceral fat burden were associated with higher levels of circulating IL-6, and this association was stronger in women than men (45).

Though obesity and BMI have been linked to CVD risk, the relationship is much more nuanced, with the pattern of fat distribution playing a role in CVD risk. BMI is unable to differentiate visceral adipose tissue (VAT) and subcutaneous adipose tissue (SAT). Visceral adiposity has been linked to CV outcomes, independent of BMI (46). Sex differences in fat distribution has been well-described, with men preferentially depositing fat in VAT regions, and females depositing fat in SAT regions during puberty (47). This effect is thought to be secondary to puberty, as estrogen promotes the deposition of fat into SAT rather than VAT. However, with age, and after menopause, the distribution of fat deposition changes in females such that that they have an increased VAT to SAT ratio, and an inflammatory profile similar to men. Increased levels of CRP, IL-6, TNF-*α* are present in VAT compared to SAT and contribute to systemic inflammation. Though pre-menopausal women have a generally lower risk of CVD than men, a study found that differences in CRP between men and women may be attributed by subcutaneous fat, and pre-menopausal women in in this cohort had higher rates of CRP than men due to the accumulation of subcutaneous fat (48). In a sub-analysis of the Framingham Heart Study, VAT had higher associations with CV outcomes than did BMI (49). This finding is also supported by the Jackson Heart Study in African Americans and the Multi-Ethnic Study of Atherosclerosis (MESA study) (50, 51).

There is abundant evidence to support that metabolic syndrome and obesity mediate increased CVD risk, in part, *via* increased systemic inflammation in women. These studies clearly support that reducing BMI would have a favorable effect on CVD-related events.

#### Physical inactivity

Physical inactivity and the metabolic syndrome and obesity are inherently related, but a few studies have specifically examined the effects of reversing physical inactivity on inflammatory biomarkers. In a Finnish study, 3,803 adults were evaluated for effects of physical exercise on CRP levels. After adjustment for age, physical activity and CRP levels were inversely associated for both men and women. However, after adjustment for all other factors, this relationship was present only for women (52). Contrasting this finding are the results from the PRINCE and CHIANTI studies, where there was an inverse relationship between fitness and CRP for men, but this finding was not seen in women after adjusting for BMI (53, 54). The reason for this discrepancy is unclear, but it may be that in the latter two studies, women's level of physical activity was not enough to decrease BMI, and ultimately, decrease CRP.

#### Hypertension

Hypertension tends to be lower in prevalence in premenopausal women compared to men of similar age. However, inflammation that occurs secondary to hypertension may increase the risk of CVD in younger women; in the aforementioned MESA study, the increased risk of hypertension with obesity was also associated with higher levels of CRP in women (55). There is limited data associating hypertension with inflammatory markers, however this association should be further explored in pre-menopausal women.

#### Dyslipidemia / diet

Diet is a critical lifestyle modification to prevent dyslipidemia and subsequent atherosclerotic disease. Inflammatory markers can be influenced by diet and exercise, but levels in women are less responsive to diet compared to men, and may be more sensitive to exercise. Bédard et al. investigated the impact of a Mediterranean diet on inflammation between the sexes. At baseline, pre-menopausal women had higher levels of high sensitivity-CRP than men (56). There was no significant difference between men and women after a 4-week diet, however, a subgroup analysis of subjects with high baseline CRP showed men had a decline in levels with the Mediterranean diet, but women did not. In another study of pre-menopausal Arab women, the hormone adiponectin was found to circulate at higher levels in serum than men, contributing to a favorable lipid profile. However, it was noted in the study that low aerobic activity may abrogate the protective effect of high levels of adiponectin on lipid profiles (57). Another study that analyzed the Pequi fruit and its ability to reduce exercise-induced inflammatory markers and blood pressure found that women had a less profound decrease in low-density lipoprotein (LDL) compared to men after Pequi oil supplementation (58). These findings of reduced sensitivity to dietary changes in women compared to men have important implications for determining which nutritional interventions would alter long-term CVD risk in women.

### Nontraditional risk factors

In this section, we review the literature available for non-traditional diseases and risk factors that may contribute to CVD risk *via* increased systemic inflammation. The data available is scarce, however, these preliminary findings warrant further investigation.

#### Baseline inflammation level and a reduced anti-inflammatory response

Studies in healthy women suggest that they have higher levels of inflammatory markers at baseline. For example, Kildey et al. investigated potential differences in the inflammatory profile of plasma donors (59). They noted that the levels of monocyte chemoattractant protein (MCP)-1, IL-12 and CRP in healthy women were higher compared to men, suggesting that at baseline, women produce higher levels of inflammatory markers. A retrospective study of 294 men and women (∼30% women) examined the relationship between bone marrow metabolism (a marker of systemic inflammation) and myocardial injury (60). Bone marrow inflammation was assessed using PET-CT, and an increase in PET-CT signal of bone marrow was observed in women with impaired myocardial perfusion compared to women with normal myocardial perfusion. No differences were observed in women. Though no systemic inflammatory marker was measured in this study, this study further supports the notion that at baseline, women may have higher levels of systemic inflammation that ultimately affect cardiovascular health. In addition, a study by Shanahan et al. found that in women, CRP increased slowly until age 15, but then there was an accelerated rate of increase until age 21, compared to men, who had a more linear increase (61).

In addition to higher levels of circulating inflammatory markers at baseline, women may have less of an anti-inflammatory response. Wegner et al. evaluated cytokine production in response to exogenous lipopolysaccharide, and found increased cytokine levels responses in women compared to men, however, there was no concomitant increase in the levels of anti-inflammatory markers in women (62). Another study of Indigenous Australian women with a high-risk profile for inflammation (based on genetic analysis) found lower circulating levels of the anti-inflammatory cytokine IL-10 (63).

Although limited, these studies highlight that woman have higher levels of inflammatory markers than men at baseline, but may have a lower anti-inflammatory response to activating (antigen) stimuli, which may contribute to increased CVD risk in women at younger ages.

#### Critical illness and sepsis

In studies of women with critical illness and sepsis, correlations of inflammation and severity of illness suggest that women have higher inflammatory responses. In a study evaluating the mortality rate between genders in a sepsis cohort, women who had sepsis were less likely to survive than males with sepsis (64). Further analysis showed that women who did not survive tended to have higher maximum CRP levels than women who did survive. Exaggerated response to infection in women compared to men has also been documented with the flu (H1N1). Studies from China of H1N1 show that men are more likely than women to become infected, but when women are infected, the symptoms are more severe, secondary to the innate and adaptive immune response (65). With the COVID-19 pandemic, more data from China emerged that women with COVID-19 disease who presented with cardiac injury had higher levels of systemic inflammatory markers, consistent with a male phenotype (66). Whether these inflammatory responses lead to cardiovascular morbidity is not known, but these findings support that women have exaggerated inflammatory responses to infection compared to men, which may increase CVD risk, especially in younger women.

#### Autoimmune diseases

Data on sex differences of CVD in autoimmune diseases is limited, but there is evidence to support that young women with autoimmune diseases are at a high risk for CVD, and studies suggest that the anti-inflammatory effects of estrogen seen in pre-menopausal women are negated by autoimmune diseases (67). Most data on autoimmune diseases and CVD risk comes from populations with rheumatoid arthritis (RA) and Systemic Lupus Erythematosus (SLE). These two conditions are known to increase CVD risk; SLE increases the risk of CVD 2.7 times that of the general population, and the risk is increased in young women with SLE (68, 69). Data associates increased inflammatory markers secondary to the disease and CVD risk. For example, disease flares of SLE can increase CRP, IL-6, and TNF-*α*, which is associated with increased triglycerides and reduced HDL levels (70, 71). In addition, patients with Rheumatoid Arthritis (RA) have a significantly higher risk of CVD compared to people without RA, and this cannot be accounted by an increased prevalence of traditional CVD risk factors in the RA group (72). In a study of 487 patients with rheumatoid arthritis, higher levels of the erythrocyte sedimentation rate (ESR) in women correlated with increased atherosclerosis (73). Since women are more likely to develop autoimmune diseases, and the risk seems to be enhanced in younger women, younger women with autoimmune disease need to be informed of and monitored for CVD (67).

#### HIV

HIV has been identified as an independent risk factor for CVD in women (74). The relative risk of myocardial infarction among women with HIV is 2.98 compared to 1.4 in men with HIV, and women with HIV in North America and Europe have higher incidences of CVD events than men with HIV (75, 76). In HIV, there is a known increase in levels of circulating inflammatory markers, which decreases after viral suppression with antiretroviral treatment. However, in women, the drop in inflammatory markers is less than that of men. In one study, even after 96 weeks of treatment and viral suppression, CRP levels remained higher in women than men (77). This discrepancy likely contributes to the disproportionate number of women with HIV who suffer from higher CVD risk despite antiretroviral treatment.

#### Osteoarthritis

Osteoarthritis is associated with an increased risk of CVD (78). This risk seems to be particularly prominent in women compared to men. A study by Perruccio et al. of men and women with arthritis found a correlation between degree of joint inflammation and CRP levels in women, but not men (79). In another study comparing sex differences between the association of osteoarthritis and CVD in men and women, CRP was the predominant contributor to overall risk of CVD in women, but not men (80). Though osteoarthritis is not considered an autoimmune disease like RA, it appears that osteoarthritis may increase CVD risk in women *via* the same mechanism as RA. However, osteoarthritis is usually a disease of older populations, so its role in CVD risk in younger women needs to be investigated further.

#### Adverse pregnancy outcomes

Adverse pregnancy outcomes—hypertensive pregnancy disorders, gestational diabetes, and preterm birth—have been found to independently increase the risk of CVD (81–83). In particular, preeclampsia (a hypertensive pregnancy disorder), increases ischemic heart disease (84). Some studies have shown that gestational diabetes increases the risk of inflammatory markers that are associated with endothelial dysfunction, but the data are scarce and there is no consensus (82). Since adverse pregnancy outcomes occur in pre-menopausal women, it is necessary to further investigate the inflammatory effects of these disease states on long-term CVD risk.

#### Hormone replacement therapy

Pre-menopausal women have lower CVD risk than age-matched men, presumably due to the cardiovascular protective effect of estrogen. However, hormone replacement therapy (HRT) with estrogen or estrogen with progesterone in post-menopausal women has not led to lower CVD risk, and instead is associated with an increased risk *via* increased CRP levels (85, 86). In a cross-sectional survey of almost 500 healthy, post-menopausal women taking HRT (mean age of 51 years), overall CRP was two times higher compared to women not on HRT (87). This relationship persisted even after controlling for BMI, age, diabetes, hypertension, hyperlipidemia, alcohol use, and cigarette consumption. Another study of women ≥65 years of age published in 1999 suggested that unopposed estrogen use was associated with 59% higher mean CRP level, and those who used estrogen and progesterone had higher levels of CRP if they also had higher BMI levels (88). Then, in 2002, results from the Women's Health Initiative were published (89). This was a randomized controlled primary prevention trial of estrogen and progestin for coronary heart disease. After approximately 5 years, the Data and Safety Monitoring Board for the study recommended stopping the trial because the incidence of invasive breast cancer, the primary adverse outcome, exceeded the stopping boundary. Though HRT is no longer the mainstay of therapy for older women, further examining the relationship between HRT and inflammatory markers will be of utmost importance in the younger, transgender population taking HRT.

#### Thyroid dysfunction

Research has shown that thyroid hormones directly influence cardiac modeling and affect CVD risk (90). Though the interaction between thyroid hormones and the cardiovascular system is complex, their direct effect on cardiovascular risk factors is mainly *via* lipid metabolism and modulation of inflammatory pathways (91). In some studies, subclinical hypothyroidism has been associated with elevated CRP levels (91). Also, the degree of thyroid hormone downregulation is associated with a high inflammatory response, with studies noting associated increases in levels of CRP, IL-6, and IL-10 (92, 93). Treatment with thyroid hormone improves CVD risk factors (90), so further research into the direct cause and effect between thyroid hormones and inflammatory markers that predispose to CVD risk is warranted.

#### Depression

Few studies looking at sex differences in circulating inflammatory cytokines of men and women have been conducted, however, depression severity has been found to correlate with levels of IL-1β and TNF-α in women (94). Compared to depressed men, depressed women have higher levels of IL-6, after controlling for BMI (94), and in a study of 231 men and women with major depressive disorder, higher CRP levels correlated with severity of depression in women but not men (95). A longitudinal analysis using data from the Midlife in the United States (MIDUS) study examined whether depressive symptoms were associated with inflammatory markers 11 years later and if there was any effect of gender on this association (96). Almost 1,000 patients where included in the follow-up analysis, who reported depressive symptoms. Among both men and women, CRP correlated with depressive symptoms, however, in women alone, IL-6 also correlated with depressive symptoms, independent of menopausal status or use of hormone replacement therapy. This study is rare in that its long-term follow-up provides insight into how a condition, such as depression, has long-term effects on inflammatory markers that predispose to CVD risk.

#### Stress

In a prospective study of 662 men and women (∼30% women) with stable coronary artery disease, after a mean 2.8-year follow-up period, 120 subjects had cardiovascular disease events (including cardiovascular death, myocardial infarction, stroke, heart failure, or unstable angina) (97). In women, higher psychological stress was associated with higher incidence of CVD events, but no association was found in men. In pre-menopausal women with CVD, markers of inflammation associated with mental and physical stress were similar to men of the same age, however, IL-6 levels persisted at a higher level after stress in women compared to men, suggesting that stress causes sustained levels of inflammation that could contribute to CVD risk in women (98). In another study looking at inflammatory markers at rest and post-stress (after a speech task), higher IL-6 and MCP-1 levels in women were associated with more cardiovascular events in women during a 3-year follow-up (99).

The relationship of acute stressor responses and systemic inflammation was also assessed in a study by Lockwood et al., of 91 healthy adults between the ages of 31–55 years (100). Thirty-three percent of participants were women. Participants completed a laboratory stress protocol with two mental stress tasks, a multisource interference task and color word task. Thirty minutes after task completion, blood samples were drawn. In men, larger stressor-evoked IL-6 levels were associated with higher CRP levels, whereas in women, larger stressor-evoked IL-6 levels were associated with lower CRP levels. This study showed there are differences in acute inflammatory responses to stress associated with CVD risk.

#### Sleep disruption

Lifestyle and social factors also increase women's risk of CVD compared to men. Assessment of sleep quality among women, using the Pittsburgh Sleep Quality Index, found that post-menopausal women who complained of waking up too early showed greater 5-year increases in IL-6, CRP, and fibrinogen as compared to males (101). In another study of 11 healthy women and 15 healthy men, monocyte cytokine production was assessed during baseline periods and after partial sleep deprivation of four hours (102). In the morning, both men and women had increased production of IL-6 and TNF-*α*, however, high levels of these inflammatory biomarkers persisted into the early and late evening the day after partial sleep deprivation in women only. Therefore, prolonged sleep disruption may increase CVD risk in women.

#### Race

The Dallas Heart Study measured CRP in White and Black subjects ages 30 to 65 and compared levels of CRP between different race and gender groups (40). After adjustment for traditional cardiovascular risk factors, estrogen and statin use, and body mass index, Black subjects and women had significantly higher levels of CRP than White subjects and men, respectively. The associations were more robust with higher CRP values (>3 mg/L). The National Health and Nutrition Examination Survey (NHANES) 1999–2000 and the Women's Health Study also found that Black women had higher levels of CRP than White women (103, 104). The NHANES study found that median CRP level in Black women was 3.5 mg/L compared to 2.5 mg/L among White women, and the CRP level was elevated independent of use of hormone replacement therapy. Though race is not a modifiable risk factor, the inflammatory profile may differ between women based on race, and further clarification on this topic, would allow for CVD risk stratification among younger women of different races.

#### Racial discrimination

Cunningham et al. investigated the association of racial discrimination and inflammation among genders (105). On average, Black women reporting 1–2 episodes of discrimination had higher levels of CRP compared to those reporting none. Similarly, White women who reported >3 episodes of discrimination had significantly higher levels of CRP than those who reported no episodes. There was no notable significance between self-reporting discrimination and CRP levels among men. Therefore, social factors can impact systemic inflammation, and ultimately, the risk of CVD in women.

#### Genetic polymorphisms

Akasaka et al. analyzed the impact of CYP2C119 polymorphisms and low-grade inflammation on coronary microvascular disorder in men and women (106). CYP2C119 polymorphisms were a predictor of coronary microvascular disorder and also associated with a higher CRP level in women compared to men. Though genetics are not modifiable risk factors, findings of how genetic polymorphisms affect systemic inflammation can account for differences in CVD risk phenotypes between younger women and age-matched men.

## Discussion

Our review of the literature emphasizes how the systemic inflammatory response in women can differ from men per disease state, increasing the risk of CVD in women for any traditional risk CVD factor. In addition, the data supporting systemic inflammation as a response to non-traditional disease states suggests that these conditions may override the protective effect of estrogen and should be taken into account when assessing the overall CVD risk in younger women. Women's risk for CVD is often underdiagnosed due to diagnostic algorithms that rely on cardiac-specific biomarkers, such as troponins (107–113).

The differences in inflammatory biomarkers identifying CVD in men and women have been explored over the last two decades. In one of the largest trials, TACTICS-TIMI, the investigators found that when women presented with unstable angina or non-ST-elevated myocardial infarction, they did not have elevations in troponin or creatine kinase as often as men (108). Instead, women had elevated markers of CRP and brain-natriuretic peptide. The latter, though, is not included in traditional diagnostic algorithms for ACS, and CRP is taken into consideration, but not part of the traditional algorithm.

In addition, when assessing for CVD risk, traditional risk calculators under- or overestimate CVD risk in women. The original algorithms were the Framingham Risk Score and the simplified Adult Treatment Panel-III (ATP-III) guidelines. A new risk estimator, the Reynolds Risk Score, was developed in 2007 to include family history of premature myocardial infarction, high-sensitivity CRP, and hemoglobin A1c (for individuals with diabetes). These factors are lacking in the Framingham Risk Score, ATP-III, and the American College of Cardiology (ACC) and American Heart Association (AHA) prediction model. In validations samples from studies with large cohorts of women, the Reynold's Risk Score demonstrates a strong predictive role for CVD and is more accurate in risk-stratifying women for a CVD event within 10 years. In contrast, the ACC and AHA prediction models overestimate risk in women, and do not yield more calibrated predictions than earlier models (114). This is significant since women are less likely to receive preventive treatment or guidance than men with similar CVD risk (7).

One reason that traditional prediction models and algorithms stratify men and women differently is due to variations in the diagnosis and treatment of pre-existing risk factors. For example, impaired fasting glucose is one of the criteria for metabolic syndrome. However, in women, it is more common to see impairments in glucose tolerance rather than impairments in fasting glucose. Since glucose tolerance is not a criterion for metabolic syndrome, women with this risk factor would not be diagnosed (7). Second, subcutaneous adipose tissue and central obesity are included in the definition of metabolic syndrome, but visceral adipose tissue in the hips and thighs, which is more common in women, is not. Last, the hormonal effects of estrogen on adipocytes is not accounted for by the definitions for metabolic syndrome. Therefore, women may be underdiagnosed for metabolic syndrome, and their CVD risk also underestimated (114). The aforementioned diagnostic algorithms have excellent ability to diagnose CVD in men, as they have been corroborated with levels of inflammatory markers found in men, however they do not incorporate inflammatory markers that are produced in response to non-cardiac stressors, which may be more important in women (115).

A complication of using inflammatory biomarkers to estimate CVD risk is that these biomarkers are not specific to any pathology. However, sex differences in circulating biomarkers that reflect distinct pathways in CVD have been identified. In a study by Lau et al., the authors measured 71 circulating CVD protein biomarkers in 7,184 participants (predominately Caucasian, 54% women, mean age 49 years) (19). In this cohort, most of the biomarkers (61, or 86%) differed significantly between men and women. Thirty-seven of the biomarkers were higher in women, including CRP. The sex differences in biomarkers were not influenced by menopausal state. This study highlights that the literature supporting sex-specific differences in systemic inflammation has important implications.

There are some limitations to our review. First, the inflammatory biomarkers and outcomes of each study were not uniform. In addition, many of these studies have small sample sizes and lower representation of women compared to men. Accordingly, the menopausal status of women in most of the studies is not specified. Therefore, it is difficult to interpret the role that hormones have on inflammation and conclude the risk of women before and after menopause. Finally, the distribution of standard markers in healthy participants is not well defined. Therefore, larger prospective studies on this topic are required for further understanding.

In conclusion, our review demonstrates that inflammatory responses in women are distinct from men and may contribute to an overall higher CVD risk, especially in younger women. In addition, review of the literature suggests that the current algorithms to quantify CVD risk in women may be inadequate, as they do not take into account sex-specific responses to diseases states that increase CVD risk. Therefore, larger prospective clinical trials with adequate women representation and specification of menopausal status are necessary to gain more insight on how to quantify and prevent CVD risk in women.
